# The Use of Platelet-Rich Plasma in Wildlife Veterinary Medicine

**DOI:** 10.3390/ani15223352

**Published:** 2025-11-20

**Authors:** Manuel Fuertes-Recuero, Teresa Encinas Cerezo, Pablo Morón-Elorza

**Affiliations:** 1 Complutense Veterinary Teaching Hospital, Complutense University of Madrid, Avda. Puerta de Hierro s/n, 28040 Madrid, Spain; manufuer@ucm.es (M.F.-R.); tencinas@vet.ucm.es (T.E.C.); 2 Department of Physiology of the Faculty of Veterinary Medicine, Complutense University of Madrid, Avda. Puerta de Hierro s/n, 28040 Madrid, Spain; 3 Department of Pharmacology and Toxicology of the Faculty of Veterinary Medicine, Complutense University of Madrid, Avda. Puerta de Hierro s/n, 28040 Madrid, Spain; 4 Fundación Oceanografic of the Comunitat Valenciana, Carrer d’Eduardo Primo Yúfera, 1, 46013 Valencia, Spain

**Keywords:** wild animal, exotic, wounds, regenerative medicine, tissue healing, orthopaedics, conservation medicine

## Abstract

Platelet-rich plasma (PRP), an autologous blood product concentrated with platelets and their associated growth factors, has been the subject of increasing investigation in veterinary medicine. Although it is widely used in domestic species, its use in wildlife is less well-explored, despite its potential to address complicated clinical scenarios such as traumatic injuries, chronic wounds, and orthopaedic conditions, which are frequently encountered in wild animals under human care. Expanding the evidence base for PRP in non-domestic species could therefore significantly advance clinical outcomes in rehabilitation, zoo medicine, and conservation initiatives. The aim of this scoping review was to identify and describe the existing evidence on the clinical use of PRP in wildlife. This included details on protocols, outcomes, species-specific considerations, and knowledge that is still missing. A literature search was performed using the databases PubMed, Scopus, Web of Science, and Google Scholar up to August 2025. Studies were eligible for inclusion if they reported on the preparation, application, or clinical outcomes of PRP in non-domestic animal species. This included case reports, research articles, and reviews. A total of 65 studies were finally included. The data were classified by species group (e.g., reptiles, birds, mammals) and clinical application (e.g., wound healing, orthopaedics, chronic disease management). The search identified a limited but growing body of evidence, primarily consisting of case reports and small-scale experimental studies. The application of PRP was found to have beneficial effects on wound closure, inflammation reduction, and enhanced tissue regeneration across multiple taxa. However, substantial variation existed across protocols in platelet concentration, activation methods, and application routes across protocols. Species-specific challenges, such as limited blood volume and logistical constraints in wildlife settings, were frequently noted. Current evidence suggests that PRP is a promising therapeutic tool in wildlife medicine; however, the development of standardised methodologies and the implementation of controlled clinical trials remain urgently needed. Widening its use could not only enhance animal welfare but also reinforce conservation initiatives and advance One Health strategies by generating insights with direct relevance to human medicine.

## Simple Summary

Wildlife veterinarians are increasingly exploring the use of platelet-rich plasma, a blood component that carries natural signals to help tissues repair themselves. This scoping review maps its use in non-domestic animals, from reptiles and birds to marine and terrestrial mammals, and highlights what remains unknown. Reported applications include closing difficult wounds, supporting bone and tendon repair, and reducing excessive inflammation. Many case reports describe faster healing and improved tissue quality with minimal side effects. However, most studies have involved small numbers of animals, and the ways in which the product is prepared and applied have differed widely. In small or endangered species, collecting enough blood can be challenging, so alternative methods are sometimes considered. To improve clinical care and facilitate meaningful comparison of results across studies, future research should employ clear, consistent methods, specify the composition of each preparation, and assess safety and efficacy through rigorously designed clinical trials involving different species. These steps will lead to better outcomes in the fields of rehabilitation, zoo medicine and conservation.

## 1. Introduction

Platelet-rich plasma (PRP) is a concentrate derived from autologous blood that is enriched with platelets and their associated growth factors. It was originally developed as a therapy to support other treatments in human medicine. Studies have shown that it can speed up wound healing, control inflammation and encourage tissue regeneration. This is achieved by releasing various bioactive molecules, including those produced by platelets [1,2,3,4]. Some of these molecules are platelet-derived growth factor (PDGF), transforming growth factor-β (TGF-β), vascular endothelial growth factor (VEGF), and insulin-like growth factor (IGF). PRP is particularly appealing for clinical use because the procedure is minimally invasive and uses autologous material, as this reduces the risk of immune rejection and pathogen transmission [2,4].

Over the past two decades, PRP in veterinary medicine has grown considerably, and its efficacy has been shown in the management of wounds, orthopaedic injuries, and chronic or refractory conditions in domestic species such as horses, dogs, and cats [4,5,6,7]. In contrast, the use of PRP in wildlife medicine is still in its early stages, with most research consisting of case reports and small-scale studies. However, there is a pressing need for effective regenerative therapies in wildlife. The animals that are under human care in zoos, rehabilitation centres and conservation programmes often present or can develop traumatic injuries, fractures and chronic wounds that are difficult to treat using conventional medical methods [8,9].

The use of PRP in wildlife animals offers opportunities, but also presents challenges [10]. On the one hand, PRP provides therapy that is tailored to the biology of the individual animal. This may enhance animal welfare, reduce recovery times, and support conservation objectives. In light of these considerations, a structured assessment of the available evidence is necessary in order to map current knowledge, identify gaps, and inform future research. This scoping review therefore aims to examine the published literature on the use of PRP in wildlife species, highlighting preparation methods, clinical indications, reported outcomes and research needs. This review aims to provide a comprehensive overview of PRP applications in wildlife medicine and discuss their implications, thereby bridging the gap between veterinary medicine in domestic animals and human regenerative medicine with wildlife animals.

## 2. Materials and Methods

A comprehensive literature search was performed to identify published evidence on the use of PRP in wildlife medicine in four databases: PubMed, Web of Science, Scopus, and Google Scholar, which were systematically searched up to August 2025. The search strategy consisted of the use of the keyword “platelet-rich plasma” AND (“wildlife” OR “non-domestic species” OR “exotic animals” OR “zoo medicine”) AND (“wound healing” OR “orthopedic injuries” OR “fracture management” OR “chronic disease” OR “oral disease”) AND (“regenerative medicine” OR “tissue repair” OR “healing outcomes”). To ensure comprehensive coverage of the available literature, the search was supplemented by manual screening of the reference lists from all eligible studies as well as from relevant review articles identified during the initial search process.

Studies were considered eligible if they reported the preparation, characterization, or clinical use of PRP in non-domestic animal species. Eligible designs included case reports, case series, experimental models, observational studies, and narrative reviews that provided original data. Publications were restricted to English and Spanish. Studies focusing exclusively on veterinary domestic species, conference abstracts without full-text availability, and articles addressing haemocomponents other than PRP were excluded.

Titles and abstracts retrieved from the database search were screened independently by two PhDs in veterinary medicine. Full-text articles that met the inclusion criteria were then assessed in detail. Discrepancies in study selection were resolved through consensus. For each included study, data were extracted on authorship, publication year, country, species investigated, number of animals, clinical condition addressed, PRP preparation and activation protocol, route of administration, treatment outcomes, and follow-up duration.

**Figure 1:**
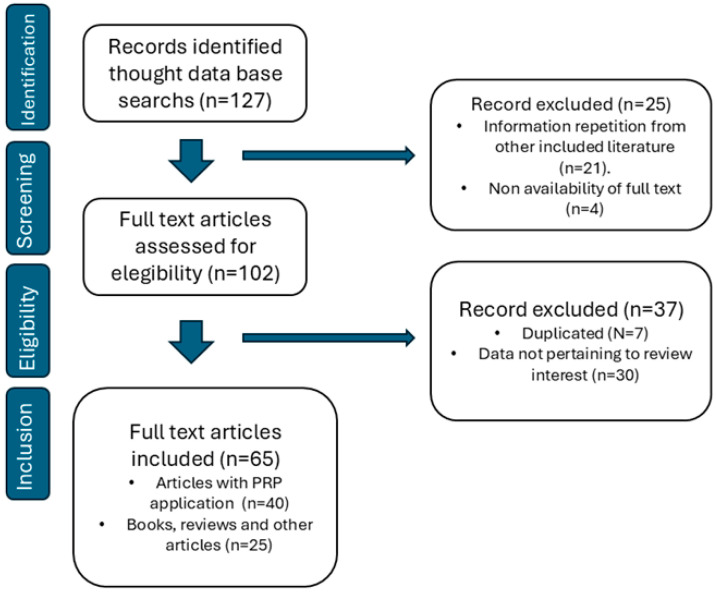
Flow diagram of the search strategy undertaken in the review.

Extracted data were synthesised narratively and grouped into species categories, namely reptiles, birds, fish and mammals. Within each category, studies were further organised according to clinical application, including wound management, orthopaedic and musculoskeletal interventions, and treatment of chronic or oral diseases. A tabular summary was prepared to provide a comparative overview of the available evidence, highlighting the diversity of protocols, clinical outcomes, and limitations identified in the current body of literature. Figure 1 shows the tracking of the information search.

## 3. Results

The search strategy yielded a total of 127 articles from the databases. During the initial screening, 21 were excluded for information repetition from other included literature, and 4 were excluded because the full text was not available, resulting in 25 exclusions in total. In the second screening, a further 37 articles were removed as they did not meet the inclusion criteria and focused exclusively on domestic species. Ultimately, this scoping review retained 65 studies for analysis, focusing on the use of PRP in wildlife. Of these, 40 were articles detailing the clinical application of PRP in wildlife, while 25 were books, reviews, and other articles containing important information about the use of PRP in wildlife.

The systematic search revealed a modest yet expanding literature on the clinical application of PRP in wildlife. Most of the studies were case reports or small experimental trials, and the majority focused on mammals, followed by birds and reptiles. The applications focused primarily on wound management, orthopaedic repair, and chronic or oral diseases. Platelet concentrates used in veterinary medicine include autologous products and, in specific contexts, allogeneic/heterologous products. They also comprise multiple formulations, such as pure PRP (P-PRP), leukocyte-rich PRP (LR-PRP), and fibrin-based derivatives like PRF. These differ in terms of their cellular content (platelet/leukocyte) and fibrin content, as well as their growth factor concentration (e.g., PDGF, TGF-β, VEGF, bFGF and IGF). Preparation typically relies on single- or double-spin centrifugation of anticoagulated whole blood, with significant variation in g-forces, times, anticoagulants and activators (CaCl_2_, thrombin…), resulting in liquid or gel formats [4,10,11]. This methodological heterogeneity and frequent under-reporting of composition make cross-study comparisons and standardisation difficult. In non-mammalian taxa (birds, reptiles…), thrombocyte-based concentrates (e.g., thrombocyte- or leukocyte-rich plasma) are adapted to the biology of the species in question and to field conditions [12,13,14]. These differences in technique and product necessitate explicit protocol reporting and species-specific characterisation to interpret efficacy and guide clinical use.

The evidence base for articles on the clinical application of PRP varies across taxa: reptiles (*n* = 5), birds (*n* = 3), fish (*n* = 1), aquatic mammals (*n* = 4), and terrestrial mammals (*n* = 27). This reflects disparities in case availability, clinical logistics, and reporting standards. Although the findings were consistently positive, variability in preparation protocols, platelet or thrombocyte concentration and activation methods limited comparability between studies.

### 3.1. Reptiles

PRP therapy has demonstrated notable regenerative effects in these taxa. In chelonians, Di Ianni et al. (2015) showed that thrombocyte-leukocyte-rich plasma (TLRP) significantly accelerated the healing of shell and skin lesions, reducing fibrosis and supporting the regeneration of functional scutes [13]. In a green bush rat snake (*Gonyosoma prasinum*), the application of TLRP topically after surgical debridement facilitated complete wound closure within 21 days, with pronounced granulation tissue formation and minimal risk of infection [15]. Similarly, a green tree python (*Morelia viridis*) treated with a multimodal protocol combining surgical repair, low-level laser therapy (LLLT), and TLRP exhibited substantial wound contraction within 18 days and reduced scarring, underscoring the synergistic potential of PRP with adjunctive therapies [16]. Repeated applications have also been effective for oral lesions such as chronic stomatitis, with significant mucosal restoration and decreased reliance on systemic antimicrobials in other reptile species [12,17]. A recent case report on the rehabilitation of a green sea turtle (*Chelonia mydas*) described the use of perilesional and intralesional injections of gelified TLRP after surgical debridement. This resulted in steady wound improvement over two months without any adverse effects [18]. Studies assessing the relation between the application of PRP in reptiles are summarised in Table 1.

### 3.2. Birds

The application of PRP, more accurately described as thrombocyte-rich plasma due to the nucleated nature of avian thrombocytes, has primarily been investigated in avian species in the context of trauma-related and chronic wound management. One of the most illustrative clinical reports is the treatment of a severe cervical wound in a greylag goose (*Anser anser*) with autologous thrombocyte-rich plasma combined with a fibrin mesh. This resulted in near-complete wound closure within 25 days, as well as preservation of the structure of the feather follicles, which are critical for thermoregulation [14]. Fernandes et al. (2019) described the successful preparation of leukocyte- and thrombocyte-rich fibrin membranes in chickens, indicating the feasibility of producing bioactive scaffolds for soft tissue repair in avian patients [19]. Similarly, PRP has been used to treat pododermatitis (bumblefoot), a condition characterised by chronic plantar ulceration and inflammation in captive birds. In one case involving a mute swan (*Cygnus olor*), the local administration of platelet- and leukocyte-rich plasma promoted granulation tissue formation and significantly reduced lesion recurrence [20]. Studies assessing the relation between the application of PRP in birds are summarised in Table 1.

### 3.3. Fish

The application of PRP in fish species, particularly teleost fish, remains a scarcely explored field, mainly due to physiological and anatomical peculiarities that challenge standard protocols.

Although clinical applications are still uncommon, experimental research is providing important groundwork. Fleming et al. (2008) showed that recombinant human platelet-derived growth factor (rhPDGF-BB) can be used to treat head and lateral line erosion syndrome (HLLES) in ocean surgeonfish (*Acanthurus bahianus*), a condition that is often seen in reef fish maintained under human care [21]. They found that a single topical application of becaplermin (Regranex^®^) significantly improved lesion healing, particularly in fish housed in non-pathogenic aquatic systems. Growth factor-based treatments can be further enhanced by modulating environmental parameters. Readers should note that despite their popularity and frequent care in aquariums and research facilities, there are currently no published studies with PRP in elasmobranchs. Studies assessing relation between the application of PRP in fish are summarised in Table 2.

### 3.4. Aquatic Mammals

The therapeutic use of PRP in aquatic mammals is still under development, although existing reports highlight its potential to promote tissue regeneration and improve ophthalmic outcomes. Simeone et al. (2018) described in bottlenose dolphins (*Tursiops truncatus*) that combining autologous PRP with mesenchymal stem cell therapy improved outcomes for corneal keratomycosis by enhancing re-epithelialisation and diminishing the inflammatory response [22]. Similarly, Canales et al. (2017) found that peri-lesional PRP injections into surgically debrided wounds accelerated granulation, minimised necrosis and reduced the need for intensive daily wound care [23]. Griffeth et al. (2014) also demonstrated PRP’s regenerative capacity, showing its ability to increase the proliferation and activity of adipose-derived mesenchymal stem cells in dolphins in vitro [24].

Recent advances in protocol optimisation strengthen the scientific foundation of this field. Morón-Elorza et al. (2021) developed a centrifugation protocol for South American sea lions (*Otaria flavescens*) that achieved a 4.7-fold increase in platelet concentration from minimal volumes of citrated whole blood. The optimal one-step protocol (900 rpm for three minutes) and a two-step method (900 rpm for three minutes followed by 2000 rpm for six minutes) preserved platelet integrity and consistently yielded high concentrations [8].

Studies investigating the application of PRP in aquatic mammals are summarised in Table 2.

### 3.5. Terrestrial Mammals

The use PRP in terrestrial mammals has been increasingly documented across a wide range of species and clinical scenarios, particularly in wound healing and orthopaedic interventions. Despite anatomical and haematological variations, both autologous and heterologous PRP formulations have consistently exhibited regenerative potential, prompting their incorporation into veterinary protocols for wildlife. Studies assessing the relationship between the application of PRP in terrestrial mammals are summarised in Table 3.

PRP has been extensively studied in small mammalian species such as rabbits, rats and mice under controlled experimental conditions. Abegão et al. (2015) showed that a heterologous PRP gel promoted granulation tissue and epithelial regeneration in full-thickness dermal wounds in rabbits compared with untreated controls [25]. Aragón-Urrego et al. (2018) also reported improved vascularisation and reduced necrosis in PRP-treated random-pattern skin flaps, indicating better flap survival [26]. In rats and mice, applying PRP to muscle contusions promoted organised myofibre regeneration and mitigated fibrosis [27]. Across rabbit models, PRP has demonstrated benefits in joint, ocular, cutaneous, and intervertebral disc healing. PRP gel outperforms platelet-rich fibrin (PRF) in corneal repair; platelet-rich growth factor (PRGF) combined with adipose-derived stem cells (ASCs) accelerates cutaneous wound healing, and leukocyte-poor PRP enhances intervertebral disc regeneration [28,29,30,31,32,33]. In guinea pigs, repeated intra-articular PRP has demonstrated chondroprotective effects, and combining PRP with low-intensity pulsed ultrasound (LIPUS) yields superior structural and histological repair, supporting a potential disease-modifying role [34,35]. In rats, a PRP-ferulic acid intradiscal hydrogel enhances extracellular matrix restoration in models of intervertebral disc degeneration [36]. Meanwhile, frozen-thawed PRP performs comparably to fresh PRP in tendon healing, enabling practical multi-dose treatment strategies [37], and PRP therapy has been reported to promote leak closure following sleeve gastrectomy [38].

Bharathidasan et al. (2015) reported a case of a blackbuck (*Antilope cervicapra*) with a tibial fracture that was treated with a combination of internal plate-rod fixation and hydroxyapatite seeded with PRP [39]. Radiographic union and restored functional mobility were achieved within eight weeks, highlighting the role of PRP in enhancing osteoconductivity and fracture repair. Similarly, Hsu et al. (2023) treated with repeated peri-lesional autologous PRP injections a perineal wound in a young Formosan sambar deer (*Rusa unicolor swinhoei*) caused by excessive maternal grooming [40]. Complete tissue regeneration and rapid granulation occurred within 20 days without the need for surgical debridement, suggesting a robust trophic and anti-inflammatory effect in cervids.

The endangered Formosan pangolin (*Manis pentadactyla pentadactyla*) has also benefited from regenerative therapies. Chen (2021) applied modified Choukroun’s platelet-rich fibrin (PRF) to deep skin wounds, resulting in significantly faster closure rates and reduced secondary infections when compared to standard wound care [41]. These findings were corroborated by Chen and Deem (2019), who documented the successful use of PRF in managing intranasal abscesses in the pangolin [42].

Wouters et al. (2015) described the topical application of PLTfix^®^, a blood-derived platelet-rich plasma (PRP) formulation enriched with dimethyl sulfoxide (DMSO), to treat extensive integumentary injuries and horn lesions in elephants (*Loxodonta africana* and *Elephas maximus*) [43]. They achieved rapid granulation, re-epithelialisation and keratin regrowth within four weeks, with no reported complications.

The application of PRP has garnered growing interest in less commonly studied domestic and farm mammals, particularly South American camelids and other large herbivores. Semevolos et al. (2016) compared two PRP preparation methods and two activation techniques in llamas and alpacas, revealing both to be feasible, though platelet and leukocyte yields varied significantly depending on the protocol [44].

**Table 3:** Summary of clinical applications of PRP in terrestrial mammal species.

Species Examples	Clinical Application	Type of Application	PRP Protocol	Main Conclusion	Reference
Rabbit (*Oryctolagus cuniculus*)	Skin flaps	Subcutaneous infiltration at the flap	Autologous PRP	Improved flap viability and vascularization	[26]
Rabbit (*Oryctolagus cuniculus*)	Dermal wounds	Topical PRP gel applied	Heterologous PRP gel	Enhanced healing; epithelialization and collagen deposition	[25]
Rabbit (*Oryctolagus cuniculus*)	Haematological validation	Not applicable (haematological validation)	Pure PRP (P-PRP)	Effective manual method for PRP in rabbits	[45]
Rabbit (*Oryctolagus cuniculus*)	Calvarial bone defects	PRP-seeded into the calvarial defect	PRP + HA-β-TCP scaffold	Enhanced osteoconduction and defect healing	[46]
Rabbit (*Oryctolagus cuniculus*)	Knee osteoarthritis (surgery-induced model)	Intra-articular injections	Autologous PRP	PRP+ ozone prevented cartilage destruction, improved weight-bearing symmetry, and improved type II collagen	[28]
Rabbit (*Oryctolagus cuniculus*)	Orthopedic cartilage/bone repair	Repeated intra-articular and intra-osseous infiltrations; intradiscal injection (per protocol)	PRGF; repeated infiltrations at predefined intervalsdouble-spin	Improved repair quality, elastic fiber organization	[32]
Rabbit (*Oryctolagus cuniculus*)	Cartilage regeneration	In vitro supplementation of culture medium with inactive PRP	Inactive PRP in culture medium; combined with TGF-β1 transfection	RP plus TGF-β1 enhanced and accelerated chondrogenic differentiation of rabbit dental pulp–derived MSCs	[31]
Rabbit (*Oryctolagus cuniculus*)	Veterinary ophthalmic surgery (corneal repair)	Topical corneal application of PRP gel	Autologous PRP gel	PRP gel was safe and promoted angiogenesis with subsequent vessel regression and faster clarity	[29]
Rabbit (*Oryctolagus cuniculus*)	Dermal wound healing/soft-tissue regeneration	Topical application and perilesional infiltrations	PRGF (plasma rich in growth factors) topical/infiltration	PRGF significantly accelerated epithelialization, collagen deposition and angiogenesis, reducing inflammation	[30]
Rabbit (*Oryctolagus cuniculus*)	Intradiscal biologic for degenerative disc disease	Intradiscal injection	Autologous Lp-PRP (leukocyte-poor) vs. Lr-PRP (leukocyte-rich)	Lp-PRP produced superior structural and histologic disc outcomes vs. Lr-PRP at mid-term	[33]
Mouse (*Mus musculus*)	PRP	Intravenous injections	Osteonecrosis	Stimulated angiogenesis and tissue repair	[47]
Mouse (*Mus musculus*)	Autologous PRP	PRP-loaded hydrogel scaffold applied to cutaneous wounds	Skin wound healing	PRP hydrogel scaffold enhanced angiogenesis and tissue repair	[48]
Mouse (*Mus musculus*)	Autologous PRP	Intramuscular injection in a contusion model	Muscle contusion injury	Intramuscular injection of PRP in rat model of contusion	[27]
Rat (*Rattus norvegicus*)	Autologous PRP	Topical PRP gel applied post-debridement	MRSA-infected surgical wounds	Topical PRP gel used post-debridement, improved healing vs. controls	[49]
Rat (*Rattus nor-ve-gicus*)	Spine—disc regeneration	Intradiscal injection	Composite hydrogel containing PRP + ferulic acid (FA); intradiscal injection	PRP–FA hydrogel showed the highest growth-factor release, good cytocompatibility, and superior restoration of disc matrix	[36]
Rat (*Rattus norve-gicus*)	Gastric surgery and leak management	Perilesional injections at gastric leak margins	MSCs and autologous PRP; injected perilesionally at leak edges	Accelerated mucosal regeneration and fibrosis/remodelling at the leak site	[38]
Rat (*Rattus norve-gicus*)	Calcaneal (Achilles) tendon transection model	Single intralesional injection	Treatment with fresh vs. frozen autologous PRP; single 50 µL intralesional injection 2 h post-op	Fresh PRP showed higher ultimate tensile strength at day 20, but groups converged by day 40, supporting frozen PRP as a practical option when multiple injections are needed	[37]
Guinea Pig (*Cavia porcellus*)	Knee osteoarthritis	Intra-articular PRP injections	PRP vs low-intensity pulsed ultrasound (LIPUS); intra-articular PRP, daily LIPUS	PRP improved boundary lubrication; LIPUS produced superior improvements in cartilage mechanical properties; both modalities beneficial	[35]
Guinea Pig (*Cavia porcellus*)	Primary knee osteoarthritis	Serial intra-articular injections	Allogenic PRP (double-spin); serial intra-articular injections	PRP reduced chondrocyte apoptosis and increased aggrecan; histological chondroprotection at mid-term follow-up	[34]
Armadillo (*Chaetophractus Villosus*)	Autologous PRP	Not applicable (no clinical administration)	Not specified (haematologic characterization)	Morphological analysis of platelets to explore regenerative potential	[50]
Pangolin (*Manis pentadactyla*)	Modified PRF	Topical application	Traumatic wounds (spine and limbs)	Topical PRF applied, reduced healing time and infection risk	[41]
Blackbuck (*Antilope cervicapra*)	Autologous PRP + Hydroxyapatite	PRP-seeded hydroxyapatite graft implanted at the fracture site with plate-rod fixation	Orthopedic fracture (tibia)	PRP-seeded scaffold with plate-rod fixation, radiographic union at 8 weeks	[39]
Formosan sambar deer (*Rusa unicolor swinhoei*)	Autologous PRP	Repeated perilesional injections	Perineal wound (maternal over-grooming)	Perilesional PRP injections, rapid closure in 20 days	[40]
Donkey (*Equus asinus*)	Autologous PRP	Intrauterine infusion during estrus	Endometritis/endometrosis	Simple centrifugation method to treat uterine inflammation	[51]
Llama (*Lama glama*) and Alpaca (*Lama alpacos*)	Autologous PRP	Not applicable (no clinical administration)	Standardization and characterization	Comparison of PRP preparation and activation methods	[44]
Elephant (*Loxodonta africana* and *Elephas maximus*)	Autologous PRP-based product	Topical application of PRP-based product (PLTfix^®^)	Complex skin lesions (foot pad, trunk)	PLTfix^®^ with DMSO led to rapid granulation and horn regrowth	[43]
Pig (*Sus domesticus*)	Autologous PRP	Topical PRP gel applied to debrided burn wounds	Partial-thickness burns	PRP used in burn healing model, assessed for re-epithelialization	[52]

## 4. Discussion

Consistent with PRISMA-ScR guidelines, this review did not include a formal risk of bias, as such evaluations are not required for scoping reviews [53]. Furthermore, given that the majority of included studies were case reports or case series, the findings should be interpreted with caution, acknowledging the inherent limitations in generalizability and methodological rigor of these study designs. Despite all this, the information found may be of great interest to veterinarians of non-domestic animals interested in applying PRP therapy.

The therapeutic use of PRP in non-domestic species has grown considerably in recent years, achieving promising results across a variety of species. In fact, 75% of articles describing the clinical use of PRP in wild animals have been published in the last 10 years. This scoping review shows that, as in domestic species such as dogs and cats [4,11], PRP is primarily used in wildlife medicine for wound management, orthopaedic support and regenerative purposes. Furthermore, PRP has, in many cases, reduced the need for surgical interventions, accelerated recovery times, and improved tissue integrity. These studies have been conducted on animals from various taxa, ranging from fish to mammals, most of which are usually found in conservation or zoological contexts. Analysis of the studies found reveals benefits that go beyond the clinical use of PRP, as well as limitations in its use, and opens guidelines for further research into the medical possibilities of this therapy in non-domestic animals.

### 4.1. Benefits of Using Platelet-Rich Plasma in Non-Domestic Animals

#### 4.1.1. Clinical Use

All reports consistently describe an improvement in wound closure rates; some of them also report increased neovascularisation and reduced infection risk, highlighting the potential as a biologically active, low-risk adjunctive therapy in wildlife rehabilitation and conservation medicine [8,30,40].

The benefits of using PRP in non-domestic animal species are particularly significant in certain situations, such as the treatment of certain conditions in reptiles (traumatic injuries or shell fractures (in the case of chelonians), as well as chronic mucosal disorders, which are common and difficult to recover from due to the slow metabolism and species-specific immune responses [12,16,54]. Overall, the current body of evidence highlights the therapeutic potential of PRP in avian clinical practice, particularly in cases involving wound healing and chronic tissue injury. As a special case, PRP has been applied in avian species (geese, poultry, and zoo birds) suffering from trauma and podiatric conditions prone to pododermatitis, demonstrating an ability to stimulate granulation tissue and reduce recurrence of the condition. [14,19,20].

Aquatic animals, such as fish and marine mammals, represent a significant frontier for PRP therapy. Although thrombocyte biology and aquatic environments complicate its application in fish, studies involving the use of recombinant growth factors in surgeonfish suggest that PRP-like therapies could be effective in treating conditions such as head and lateral line erosion syndrome [21,55]. In marine mammals, autologous PRP has demonstrated promising results not only in the treatment of soft tissue wounds but also in ophthalmic conditions [22,23,24].

Some authors suggest the possibility of including other cells in PRP, in addition to platelets, to provide additional therapeutic benefits. Thus, when PRP is combined with stem cells, it is suggested that tissue regeneration may be improved by the synergistic action of both cell types [4,11]. Other studies suggest that the therapeutic potential of combining mesenchymal stem cells (MSCs) and PRP is particularly promising for the treatment of complex wounds, which are often complicated by infections and high mortality rates. As highlighted by Johnson (2024), the synergistic effects of MSCs and PRP can significantly enhance regenerative processes, including angiogenesis and immunomodulation [56]. The inherent antibacterial properties of MSCs further augment this approach, offering a critical advantage in managing infected or chronic nonhealing wounds, a common challenge in exotic animal and wildlife care [30]. Although direct studies in exotic species are limited, successful applications in domestic animals, such as the resolution of multidrug-resistant infections in dogs following MSC therapy, support the translational potential of this strategy for wildlife. For instance, unpublished clinical successes, such as the resolution of a chronic tail wound in an elephant treated with PRP and MSCs, underscore their use in complex cases [56].

#### 4.1.2. Wildlife Medicine Logistics

Working with some non-domestic species presents notable challenges, such as the difficulties of handling animals with large body mass or aggressive behaviour. In addition, logistical constraints can arise when treatments need to be administered outside clinical settings. These limitations can be addressed through PRP preparation systems designed for field use, such as portable centrifuges or lyophilised PRP kits [10]. These innovations offer practical solutions for in situ treatment in remote locations, field environments, or wildlife rehabilitation centres.

In marine mammals, a notable advantage of using PRP therapy is that these individuals can be trained to voluntarily provide blood samples, which reduces stress during sampling and facilitates PRP preparation. Moreover, the anti-inflammatory cytokines it contains, including IL-1Ra and TGF-β, may also promote healing in the presence of environmental stressors [22,24].

#### 4.1.3. Basic and Translational Research

With the aim of refining PRP therapies, some authors have conducted studies aimed at characterising the morphology of blood cells, shifting direct clinical applications of platelet preparations to the background. Studies using PRP in fish have helped to clarify the active role played by thrombocytes in these animals in haemostasis and immune responses, including phagocytosis, antigen presentation and cytokine release. This suggests that PRP could play a promising but currently underutilised role in regenerative medicine in fish [57]. Furthermore, insights from thrombocyte ultrastructure and gene expression (CD41, c-Mpl and GATA1) reinforce the evolutionary homology between fish thrombocytes and mammalian platelets. This validates the translation of PRP-based approaches to other species [57].

Similarly, in xenarthrans (particularly the large hairy armadillo, *Chaetophractus villosus*), studies have focused more on the characterization of blood cell morphology than on direct clinical applications of PRP. Bermúdez et al. (2004) conducted a detailed haematological investigation, describing the morphological features and relative abundance of platelets in this species and emphasising their potential role in haemostasis and wound healing [50]. So, while no therapeutic interventions using PRP have yet been documented in this species, this foundational understanding of their thrombocyte biology could inform the future development of regenerative therapies within this taxonomic group.

In two of the articles consulted, the main objective is more technical and methodological than clinical, as they explore the different ways of preparing PRP. Morón-Elorza et al. (2021) have described the optimisation of PRP for sea lions (*Otaria flavescens*) and Semevolos et al. (2016) previously published a similar article on PRP and platelet activation in llamas and alpacas [8,44].

As for translational research, rabbit studies provide valuable insights into the influence of PRP on bone regeneration, tendon repair and epithelial healing [25,26,46]. Similarly, rodent models have demonstrated PRP-mediated angiogenesis and tissue regeneration in cases of musculoskeletal injury. While these models are not wildlife per se, they are significant as translational models to evaluate the regenerative efficacy of PRP and therefore provide foundational data that can be extrapolated to free-ranging or rehabilitated lagomorphs and rodents of a similar size and physiology [30,47,48,49].

Other studies highlight the expanding therapeutic landscape of PRP beyond traditional companion and farm animals. Although pigs are less frequently treated with PRP in clinical veterinary settings, they have served as a valuable translational model for wound healing studies. Singer et al. (2018) described that PRP significantly enhances epithelial regeneration and reduces healing time in a porcine model of partial-thickness burns [52]. This reinforces the relevance of swine physiology in validating PRP-based interventions. In donkeys, Fantini et al. (2021) successfully used PRP for intrauterine infusion in cases of chronic endometriosis and showed promise as a regenerative therapy in reproductive medicine, suggesting its applicability to other tissues [51].

### 4.2. Limitations on the Use of Platelet-Rich Plasma in Non-Domestic Animals

Although the review of PRP therapy in non-domestic animals demonstrates considerable potential, its practical application is limited by several factors. Some of these limitations are related to the morphological and physiological characteristics of the targeted species, while others relate to the preparation method used.

The small blood volume and limited access to donor sites in some small individuals, like some species of birds, amphibians, reptiles and fish, pose practical limitations for PRP harvesting [58], highlighting the need for simplified single-spin protocols and further validation studies tailored to these species. Notably, the relatively small amount of blood that can be safely collected from small birds necessitates adapted methodologies. Although single-spin centrifugation protocols using minimal volumes have been proposed using chickens as a model (*Gallus domesticus*) [58], concerns remain regarding the resulting thrombocyte concentration and the reproducibility of such preparations. In marine mammals, limited venous access, minimising stress and maintaining the integrity of samples in the aquatic environment are the main limitations of PRP therapy [8].

One major technical limitation for PRP application in non-domestic animals is the distinctive nature of blood cells in some animal species. In reptiles, PRP use remains limited due to haematological differences, particularly the nucleated nature of thrombocytes and the low baseline count [59]. Fish thrombocytes differ in morphology and origin from those of other species; they are also nucleated and originate from thrombocyte/erythrocyte progenitor cells (TEP) in the pronephros and mesonephros, rather than from megakaryocytes as in mammals [57]. In elasmobranchs, a granulated thrombocyte containing cytoplasmic granules that stain with Giemsa has been identified in several shark species, particularly within the family Carcharhinidae; however, the functional significance of these granules remains unknown [60]. Furthermore, as Mudroňová et al. (2014) have highlighted, the haematological characteristics of avian species, including the difficulty in distinguishing between mononuclear cells and thrombocytes based on morphology, complicate the standardization of PRP formulations and the quantification of their cellular components [61].

Logistical and ethical limitations in obtaining sufficient volumes of autologous blood in small or critically endangered species, together with the difficulty of handling aggressive or dangerous animals, underscore the need to explore safe and effective alternatives, such as allogeneic or xenogeneic sources of PRP, which carry a risk of possible immunogenic reactions [5]. However, the second major limitation mentioned (the large differences in blood cell morphology between species) restricts the practical implementation of this approach.

Another significant limitation is the lack of standardisation in preparation methodologies; studies differ considerably in terms of the use of anticoagulants, centrifugation speeds, centrifugation cycles (number of cycles and duration), activating agents, different growth factors, and the presence or absence of leukocytes. This results in significant heterogeneity in the final products [9,10]; even when using identical protocols, disparities in operator technique can result in substantial variations in platelet concentration and leukocyte content [9,11]. Furthermore, the absence of minimum reporting requirements and quality control parameters, such as platelet enrichment factors and pH stability, compromises clinical reproducibility [62]. Many of the revised studies use different PRP formulations, including autologous and heterologous sources, which makes it difficult to compare the results.

As described in domestic species by Reyes et al. (2024), a “one-size-fits-all” approach to centrifugation parameters is ineffective, as platelet recovery and yield vary considerably even between closely related mammalian species due to differences in platelet characteristics, haematocrit, and baseline platelet counts [10]. This variability is amplified across the diverse taxa encompassed in wildlife medicine, from reptiles with nucleated thrombocytes to birds with high metabolic rates and fish with unique thrombocytic biology. Therefore, the significant heterogeneity in PRP preparation protocols identified in this review highlights a fundamental challenge in wildlife regenerative medicine identified by many authors: the lack of species-specific optimization of PRP preparation. For example, Ortiz and Esteban (2024) emphasised that species-specific modifications to centrifugation protocols are essential for generating effective PRP fractions in fish, given variations in blood volume, viscosity, and nucleated cell profiles [55].

Finally, these wide differences between species could compromise the clinical application in non-domestic animals of translational studies conducted in other species. Some studies have revealed that the therapeutic success found in fish is similar to the mechanisms observed in other vertebrates, indicating that platelet-rich plasma (PRP), whether autologous or xenogeneic, could also promote epithelial regeneration, provided that it is adapted to the thrombocyte physiology of each species. However, the scale and clinical relevance of findings in laboratory and domestic animals (rats and mice) remain insufficiently explored in larger taxa [27,47].

### 4.3. The Future of PRP Use in Non-Domestic Animals

The consistent regenerative properties of PRP, coupled with its ease of preparation, make it a very promising tool for improving animal welfare in clinical and conservation settings [9,11,51,63]. However, the current empirical and scientific basis for PRP in wildlife medicine is limited and largely anecdotal compared to that which exists for companion animals, farm animals, and humans. Therefore, the promising future of PRP therapy in this field requires studies to provide the scientific and technical basis for its application; further research is urgently needed to standardise procedures, optimise protocols, and validate their efficacy across diverse non-domestic species [44]. Future studies may also explore emerging strategies, such as combination therapies with mesenchymal stem cells (MSCs), which have already shown promising results and have the potential to transform the management of severe or complicated wounds in exotic animal medicine.

Proposals for standardising PRP classification and reporting frameworks have been made in human and domestic animal medicine [9,10,64,65]; efforts must now focus on optimising PRP protocols in the context of wildlife, validating their safety and efficacy through robust clinical trials, under experimental conditions that account for the haematological and environmental peculiarities of each species [21,55]. The specific standardisation of these protocols will facilitate both the autologous application of PRP in clinical practice and comparisons between species and translational knowledge.

Therefore, the development of effective PRP therapies for wildlife must be predicated on foundational studies that establish tailored preparation methods for each species or taxonomic group to ensure predictable and therapeutically relevant platelet concentrations. The recent development of minimum reporting guidelines for veterinary PRP by Sharun and Banu (2025) provides a crucial framework that should be universally adopted in future wildlife studies [9]. By mandating the consistent reporting of critical parameters such as centrifugation protocols, anticoagulant use, and final platelet and leukocyte concentrations, the adoption of such guidelines will ensure that future PRP preparations are accurately characterised and their results are reproducible. This shift is fundamental for enabling meaningful cross-study comparisons, facilitating robust meta-analyses, and ultimately transforming anecdotal evidence into a reliable, evidence-based body of knowledge for wildlife regenerative medicine.

Establishing specific methodologies for multiple species of non-domestic animals is a challenging task that can be alleviated by the use of allogeneic PRP, which also mitigates some of the logistical limitations for obtaining blood mentioned above. This practice also has other benefits, such as rigorous donor selection and the implementation of processing methods to mitigate immune rejection. Investigating these alternatives is not merely complementary but essential to expanding the scope of regenerative medicine, ensuring that the benefits of PRP are not limited by the size or conservation status of each animal.

## 5. Conclusions

This scoping review underscores the increasing attention to PRP and its promise as a regenerative therapeutic approach in wildlife medicine. Although methodological variability and insufficient scientific basis limit its interpretation, the combined analysis of available studies shows that PRP can accelerate wound healing, improve tissue regeneration, and reduce the risk of infection in a wide range of non-domestic species. PRP has been used to treat various clinical conditions in species ranging from birds and reptiles to aquatic and terrestrial mammals, often yielding positive results with few adverse effects. However, the field is still in the early stages, characterised by inconsistent reporting, protocols, and a lack of controlled trials. To advance the responsible integration of PRP into wildlife clinical practice, future research must prioritise the development of species-specific preparation guidelines, standardised classification systems, and rigorous efficacy evaluations. Incorporating haematological baselines, optimising dosage strategies, and exploring novel delivery platforms will also be essential for maximising therapeutic outcomes.

## Figures and Tables

**Table 1:** Summary of clinical applications of PRP in reptile and avian species.

Species Examples	Clinical Application	Type of Application	PRP Protocol	Main Conclusion	Reference
Reptiles					
Green Bush Rat Snake (*Gonyosoma prasinum*)	Degloving lesion	Topical application over the wound bed following surgical debridement	Single topical TLRP	Accelerated healing, reduced infection. Complete healing in 21 days	[15]
Green Tree Python (*Morelia viridis*)	Soft tissue wound	Topical application adjunctive to surgery and low-level laser therapy	Surgery + LLLT and Single TLRP	Accelerated healing. Healing in 18 days, minimal scarring	[16]
Red-eared slider *(Trachemys scripta elegans)*	Traumatic injuries (fractures of shell and penetrating injuries)	Perilesional injections and topical PRP gel applications	TLRP injected in some cases, and TLRP gel in others	Accelerated healing. induced the formation of a hardened tissue providing a mechanical protection against bacterial and/or mycotic contamination and tissue dehydration and desiccation	[13]
Mediterranean tortoises (*Testudo* spp.)					
Veiled chameleon (*Chamaeleo calyptratus*)	Maxillary osteolysis secondary to chronic periodontal disease	Direct intra-lesional injection into the necrotic maxillary bone	Heterologous TLRP	Accelerated healing. TLRP was obtained from a healthy conspecific and injected with a 26G syringe directly into the necrotic bone	[12]
Ball python (*Python regius*)	Chronic facial cellulitis	TLRP topical irrigation			
Green turtle (*Chelonia mydas*)	Cutaneous lesions and abscesses (jaw and forelimbs) during rehabilitation	Perilesional and intralesional injections; gelified TLRP placed into the wound bed and retained with nylon film after surgical debridement	Autologous thrombocyte-leukocyte-rich plasma	Favourable wound-healing progression over two months with no adverse reactions reported	[18]
Birds					
Greylag goose (*Anser anser*)	Severe cervical wound	Fibrin mesh sutured over the defect with local autologous thrombocyte-rich plasma	Autologous thrombocyte-rich plasma + fibrin mesh	Near-complete healing in 25 days; preserved feather follicles	[14]
Chicken (*Gallus gallus domesticus*)	Soft tissue regeneration (experimental fibrin membranes)	Topical application of leukocyte- and thrombocyte-rich fibrin membranes (experimental scaffold preparation; in vivo route not described).	Leukocyte- and thrombocyte-rich fibrin membranes	Feasible PRP preparation; potential for clinical use	[19]
Mute swan (*Cygnus olor*)	Chronic plantar pododermatitis (bumblefoot)	Single topical application to the lesion following surgical debridement	Thrombocyte–leukocyte-rich plasma (TLRP)	Granulation tissue formation; reduced recurrence	[20]

**Table 2:** Summary of clinical applications of PRP in fish and aquatic mammal species.

Species Examples	Clinical Application	Type of Application	PRP Protocol	Main Conclusion	Reference
Fish species					
Ocean surgeonfish (*Acanthurus bahianus*)	Head and lateral line erosion syndrome	Single topical application	Topical recombinant human PDGF-BB (Regranex^®^)	Improved healing; full recovery when housed in controlled aquatic systems	[21]
Aquatic mammal species					
Bottlenose dolphin (*Tursiops truncatus*)	Corneal keratomycosis	Subconjunctival injections	Autologous PRP + stem cell therapy	Improved re-epithelialization; reduced inflammation	[22]
Bottlenose dolphin (*Tursiops truncatus*)	Traumatic soft tissue wounds	Perilesional autologous PRP injections after surgical debridement	Peri-lesional autologous PRP injections	Accelerated granulation; reduced necrosis; less daily care required	[23]
Bottlenose dolphin (*Tursiops truncatus*)	Stem cell proliferation (in vitro)	in vitro supplementation of culture medium with PRP	PRP supplement in culture	Enhanced MSC proliferation and activity	[24]
South American sea lion (*Otaria flavescens*)	PRP production protocol optimization	Not applicable (laboratory protocol optimization)	Centrifugation protocol: 900 rpm × 3 min (1-step) or +2000 rpm × 6 min (2-step)	Max platelet concentration ~4.73; platelet integrity preserved	[8]

## Data Availability

The data supporting the findings of this study are available from the corresponding author upon reasonable request.

## Abbreviations

The following abbreviations are used in this manuscript:

PRP	Platelet-Rich Plasma
LR-PRP	Leukocyte-Rich Platelet-Rich Plasma
P-PRP	Pure Platelet-Rich Plasma
PRF	Platelet-Rich Fibrin
TLRP	Thrombocyte- and Leukocyte-Rich Plasma
PRGF	Plasma Rich in Growth Factors
hLLE	Homologous Lyophilised Leukocyte Extract
PDGF	Platelet-Derived Growth Factor
TGF-β	Transforming Growth Factor Beta
VEGF	Vascular Endothelial Growth Factor
bFGF	Basic Fibroblast Growth Factor
IGF-1	Insulin-Like Growth Factor 1
EGF	Epidermal Growth Factor
HGF	Hepatocyte Growth Factor
CTGF	Connective Tissue Growth Factor
MSCs	Mesenchymal Stem Cells
ASCs	Adipose-Derived Stem Cells
LLLT	Low-Level Laser Therapy
DMSO	Dimethyl Sulfoxide
BMPs	Bone Morphogenetic Proteins
IL-1Ra	Interleukin-1 Receptor Antagonist
IL-1	Interleukin 1
TNF-α	Tumor Necrosis Factor Alpha
